# Functional analysis of disease-associated polymorphism LRP5.Q89R

**Published:** 2011-04-08

**Authors:** Weiming Mao, Robert J. Wordinger, Abbot F. Clark

**Affiliations:** Department of Cell Biology & Anatomy, North Texas Eye Research Institute, University of North Texas Health Science Center, Ft. Worth, TX

## Abstract

**Purpose:**

The canonical wingless and Int1 (Wnt) signaling pathway plays key roles in multiple biologic events. The pathway co-receptor, low density lipoprotein receptor-related protein 5 (LRP5), is involved in the pathogenesis of retinal diseases and has been implicated in glaucoma. We studied whether a disease-associated polymorphism LRP5.Q89R, which is located in the second blade of the first β-propeller domain, directly alters Wnt signaling activity with cell-based assays.

**Methods:**

The LRP5.Q89R polymorphism was evaluated by transfection of HEK293T or GTM3 cells with expression vectors. LRP5 expression and interaction with the molecular chaperone mesoderm development (MESD) were determined by western immunoblotting and co-immunoprecipitation analyses. To compare membrane-associated LRP5 proteins, surface proteins were labeled with biotin and pulled down with avidin beads followed by western immunoblotting. TCF-reporter plasmid-based luciferase assays were used to determine whether LRP5.Q89R affects the canonical Wnt signaling, or has altered efficacy to suppression by Dickkopf-1 (DKK-1).

**Results:**

Cell-based assays showed that this polymorphism did not change protein expression, interaction with the molecular chaperone MESD, protein trafficking, Wnt signaling transduction, or its efficacy in DKK1-mediated inhibition.

**Conclusions:**

Our data suggest that this specific polymorphism does not appear to alter the canonical Wnt signaling pathway. Further studies of LRP5 polymorphisms are needed to elucidate their roles in various associated diseases.

## Introduction

Low density lipoprotein receptor-related protein 5 (LRP5) (GenBank: NM_002335) is a co-receptor of the canonical wingless and Int1 (Wnt) signaling pathway [1]. The canonical Wnt-β-catenin signaling pathway regulates multiple biologic events including proliferation, apoptosis, and development [2,3]. In the absence of the Wnt protein or other pathway activators, a protein destruction complex consisting of adenomatous polyposis coli (APC), Axin, glycogen synthase kinase 3 (GSK3), and casein kinase 1 (CK1) forms in the cytoplasm. This complex phosphorylates the transcription factor β-catenin, marking it for ubiquination and degradation by the proteosome. When a Wnt ligand binds to its receptor Frizzled (FZD) and co-receptor LRP5 or LRP6, a signal is transduced through Dishevelled (DVL), leading to disassembly of the protein destruction complex. Unphosphorylated β-catenin accumulates in the cytoplasm and subsequently translocates into the nucleus, where it regulates gene expression by interaction with T cell factor/lymphoid enhancer factor (TCF/LEF) transcription factors. Dysregulation of this signaling pathway has been reported in cancers, bone, and ocular diseases [4].

As a co-receptor of the canonical Wnt signaling pathway, LRP5 plays an important role in signal transduction and regulation. Its intracellular domain may be phosphorylated by CK1 and serve as a docking site for Axin, facilitating the disassembly of the Axin-APC-GSK3-CK1 protein complex [5]. The LRP5 extracellular domain, however, provides binding sites for some of the secreted Wnt pathway inhibitors. For example, secreted Wnt pathway inhibitor Dickkopf-1 (DKK-1) blocks the Wnt pathway by binding to LRP5 and disrupting the formation of Wnt-FZD-LRP5 complex [6,7].

Studies have shown that LRP5 plays key roles in eye development and eye diseases [8]. LRP5 knockout or mutant mice have persistent hyaloid vessels or incomplete peripheral retina vascularization [9,10]. These phenotypes are similar to human familial exudative vitreoretinopathy (FEVR). Genetic screening of human patients has discovered diverse LRP5 polymorphisms that are associated with these diseases [11,12]. Complete LRP5 polymorphism information is available at The National Center for Biotechnology Information, which contains more than 40 reported disease-associated LRP5 SNPs. Another helpful source of LRP5 polymorphism is available at Atlas of Genetics and Cytogenetics in Oncology and Haematology. LRP5 polymorphisms causing missense mutations which are either eye disease-related or have been biologically studied are listed in Table 1.

**Table 1:** Summary of disease-associated LRP5 polymorphisms.

cDNA change*	**Protein change**	**Associated diseases/phenotype**	**Effect on Wnt signaling**	**Biochemical phenotype**	**References**
c266A>G	Q89R	FEVR; LBM; spinal osteoarthritis	Unknown	Unknown	[20,32]
c331G>T	D111Y	HBM	Increase	Decreases Dkk1 and SOST inhibition	[26,30]
c461G>T	R154M	HBM	Increase	Decreases SOST inhibition	[26]
c511G>C	G171R	HBM	Increase	Decreases Dkk1 and SOST inhibition	[30]
c512G>T	G171V	HBM	Increase	Decreases MESD binding; decreased cell surface presentation; decreased SOST inhibition/binding	[26,28]
c593A>G	N198S	HBM	Increase	Decreases SOST inhibition	[26]
c640G>A	A214T	HBM	Increase	Decreases Dkk1 and SOST inhibition/binding	[26,30]
c724G>A	A242T	HBM	Increase	Decreases Dkk1 and SOST inhibition/binding	[30]
c758C>T	T253I	HBM	Increase	Decreases Dkk1 and SOST inhibition/binding	[26,30]
c844A>G	M282V	HBM	Increase	Decreases Dkk1 and SOST inhibition/binding	[30]
c1330C>T	R444C	FEVR	Decrease	Inhibits Norrin-FZD4 mediated pathway activation	[20]
c1481G>A	R494Q	OPPG	Decrease	Increases Dkk1 inhibition	[8,22]
c1564G>A	A522T	FEVR	Decrease	Inhibits norrin-FZD4 mediated pathway activation	[20]
c1708C>T	R570W	OPPG	Decrease	Increases Dkk1 inhibition	[8,22]
c3989C>T	A1330V	FEVR	Decrease	Inhibits norrin-FZD4 mediated pathway activation	[20]
c4574T>C#	V1525A#	BMD#	Decrease	Unknown	[34]
c4619C>T	T1540M	FEVR	Decrease	Inhibits norrin-FZD4 mediated pathway activation	[20]
c4643G>T	C1548F	FEVR	Decrease	Inhibits norrin-FZD4 mediated pathway activation	[20]

*The number starts from the first nucleotide of the coding region. #The predominant allele is controversial [34]. The SNP database recorded c4574C (1525A) as the predominant type. However, c4574C (1525A) is less capable of signal transduction compared to c4574T (1525V). HBM: high bone mineral density; LBM: low bone mineral density; OPPG: Osteoporosis-pseudoglioma syndrome.

In addition to the diseases described above, the canonical Wnt signaling pathway has recently been implicated in primary open angle glaucoma (POAG) [13]. POAG is a leading cause of visual impairment and blindness worldwide [14]. This disease is characterized by optic neuropathy and progressive, painless loss of peripheral vision. Although the exact mechanisms of POAG are not clear, clinical studies have shown that elevated intraocular pressure (IOP) is an important risk factor for developing POAG, and lowering IOP slows disease progression and protects retinal ganglion cells [15-18]. Our previous studies showed that expression of the Wnt antagonist secreted frizzled-related protein 1 (SFRP1) is elevated in the trabecular meshwork cells of POAG patients [13]. Increased expression of SFRP1 significantly elevated IOP in perfusion cultured human eyes and in mouse eyes. Topical ocular administration of a selective GSK3β inhibitor, which stabilizes β-catenin and enhances Wnt signaling activity, blocked SFRP1-induced IOP elevation in mouse eyes.

**Figure 1:**
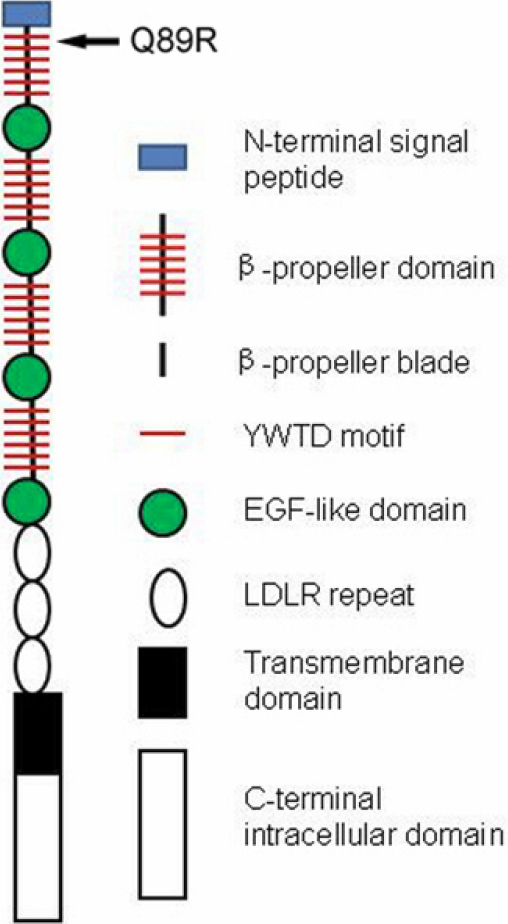
The structure of LRP5. The LRP5 protein has an intracellular domain (white rectangle), a single transmembrane domain (black rectangle), an extracellular domain containing a signal peptide (blue rectangle), four EGF-like domains (green circles) which are separated by four β-propeller domains (black vertical line and horizontal red bars), and three LDLR repeats (white ovals). Each β-propeller domain has six blades (short black bars) and five YWTD motifs (red bars). The Q89R polymorphism is located in the second blade of the first β-propeller domain (arrow).

All these findings suggest that the canonical Wnt pathway plays important roles in the pathogenesis of retinal diseases as well as glaucoma. We wanted to determine whether disease-associated Wnt gene family polymorphisms/mutations directly alter Wnt signaling activity. We first examined LRP5 due to its importance in canonical Wnt signal transduction. One polymorphism, LRP5.Q89R has been associated with eye diseases [19,20]. LRP5 protein structure is shown in Figure 1. It has an intracellular domain, a single transmembrane domain and an extracellular domain containing a signal peptide, four EGF-like domains, which are separated by four β-propeller domains and three low density lipoprotein receptor (LDLR) repeats [21]. Each β-propeller domain has six blades and five tyrosine-tryptophan-threonine-aspartic acid (YWTD) motifs [22]. The Q89R polymorphism is located in the second blade of the first β-propeller domain.

Although LRP5.Q89R is disease-associated, whether and how LRP5.Q89R affects the Wnt pathway remains unclear. We studied this polymorphism in terms of its expression, interaction with a molecular chaperone, trafficking and functionality in HEK293T cells because this cell line has been well characterized and widely used in studying the Wnt signaling pathway. Because cell signaling pathways and associated functions are often cell-type specific, we also tested LRP5.Q89R in a trabecular meshwork cell line GTM3. Our data suggest that this specific polymorphism does not alter the canonical Wnt signaling pathway.

## Methods

### Vector construction

The human DKK1 expression vector, pcDNA3.1-DKK1 was constructed by subcloning the human DKK1 coding sequence (CDs) into pcDNA3.1MycHisC (Invitrogen, Carlsbad, CA) at 5′ KpnI and 3′ NotI restriction sites, respectively. The Kozak sequence was introduced upstream of the start codon of DKK1 CDs to enhance expression [23]. The stop codon was kept so that only the native DKK1, not a fusion protein with the MycHis tag, is expressed. pcDNA3.1-hLRP5.WT-MycHisC and pcDNA3-mMESD-FLAG were kind gifts from Drs. Matthew Warman (HHMI/Childrens Hospital/Harvard, Boston, MA) and Bernadette Holdener (Stony Brook University, Stony Brook, NY), respectively. pcDNA3.1-hLRP5.Q89R-MycHisC and pcDNA3.1-hLRP5.G171V-MycHisC were constructed by mutating the pcDNA3.1-hLRP5.WT-MycHisC vector using a Site Directed Mutagenesis Kit (Agilent Technologies, Santa Clara, CA). The empty control vector, pcDNA3.1MycHisC was constructed by digesting pcDNA3.1-LRP5.WT-MycHisC with EcoRI and EcoRV and ligating the remaining ends after blunting with Klenow fragment (New England Biolabs, Ipswich, MA). All vectors were sequence-verified by Genewiz (Genewiz Inc., South Plainfield, NJ) or the DNA Core Facility at the University of Iowa, Iowa City, IA.

### Protein expression study

HEK293T/17 (ATCC, Manassas, VA) or GTM3 cells (a gift from Alcon Research, Ltd., Fort Worth, Texas) [24] were cultured with low glucose-DMEM (Thermoscientific, Worcester, MA), 10% fetal bovine serum (Invitrogen) with 1% penicilin+streptomycin and 2 mM glutamine (Thermoscientific). Four hundred ng empty vector, pcDNA3.1-LRP5.WT-MycHisC or pcDNA3.1-LRP5.Q89R-MycHisC was transfected into 4×10^5^ HEK293T/17 cells or GTM3 cells in a 12-well plate with 2.4 μl SureFECT™ transfection reagent (Qiagen, Frederick, MD). Eighteen hours after transfection, medium was changed and cells were cultured for an additional 24 h. Cells were then washed and scraped in ice-old PBS. Cell pellets were incubated with M-PER lysis buffer (Thermoscientific) plus a proteinase inhibitor cocktail (Thermoscientific) on ice. Supernatant was collected for protein estimation with the Dc Protein Assay Kit (Bio-Rad, Hercules, CA). For western blotting, 30 μg of total protein was run on an 8% SDS–PAGE gel and transferred to a polyvinylidene fluoride (PVDF) membrane. The LRP5 fusion protein was detected with a mouse anti-cMyc antibody (9E10; Santa Cruz Biotechnology, Santa Cruz, CA). Glyceraldehyde 3-phosphate dehydrogenase (GAPDH) was used as an internal loading control and was probed with rabbit anti-GAPDH (Cell Signaling, Beverly, MA). Secondary antibodies (goat anti mouse-HRP and anti rabbit-HRP) were purchased from Thermoscientific, Santa Cruz, or Cell Signaling. Antibody signals were detected using Femto substrate (Thermoscientific), and images were captured by the FluroChem imaging system (Cell Biosciences, Santa Clara, CA). Experiments were performed in biologic replicates.

### Densitometry study

To quantitate protein expression levels, the optic density of LRP5 and GAPDH protein bands were measured from the digital image by the AlphaEasyFc software (Cell Biosciences) or ImageJ (NIH, Bethesda, MD). Two normalizations were performed for statistical analysis. First, the expression of LRP5 was normalized with GAPDH. Second, the expression of LRP5.WT was set at 100% and LRP5.Q89R was then normalized to LRP5.WT.

### Co-immunoprecipitation (Co-IP) and pull-down assay

One or two μg of empty vector, pcDNA3.1-LRP5.WT-MycHisC, pcDNA3.1-LRP5.Q89R-MycHisC, or pcDNA3.1-LRP5.G171V-MycHisC was co-transfected with 1 or 0.5 ug pcDNA3-MESD-FLAG into 3.5×10^6^ HEK293T/17 cells or 2×10^6^ GTM3 cells grown in 60 mm culture dishes using 12 μl SureFECT™ transfection reagent (Qiagen). Eighteen hours after transfection, medium was changed and cells were cultured for an additional 24 h. Cells were then washed and scraped in ice-old PBS. Cell pellets were incubated with binding/wash buffer (TBS with 1% Triton X-100) plus proteinase inhibitors cocktail (Thermoscientific) on ice. After brief sonication, 100 μg total protein from each dish was incubated with 2 μg mouse anti-FLAG antibody (M2; Agilent Technologies) in a total volume of 200 μl. After 2 h incubation at 4 °C, the protein and antibody mix was incubated with 10 μl of magnetic Protein G particles (New England Biolabs) at 4 °C for 1 h. Particles were separated with a magnetic stand (Promega, Madison, WI) and washed with binding/wash buffer four times. Particles were boiled in 2× Laemmli buffer with β-mercaptoethanol for western immunoblotting. Experiments were performed in biologic replicates.

### Surface protein labeling

Protocols for labeling cell surface proteins were adapted from previously published methods [25,26]. In the 60 mm culture dish, 1 or 2 μg empty vector, pcDNA3.1-LRP5.WT-MycHisC, pcDNA3.1-LRP5.Q89R-MycHisC, or pcDNA3.1-LRP5.G171V-MycHisC was transfected into 3.5×10^6^ HEK293T/17 or 2×10^6^ GTM3 cells using 6 μl SureFECT™ transfection reagent (Qiagen). Eighteen hours after transfection, medium was changed and cells were cultured for 24 h. Cells were washed with ice-old PBS containing 1 mM MgCl_2_ and 1 mM CaCl_2_. After a PBS wash, 0.5 ml of Sulfo-NHS-LC-Biotin (0.5mg/ml in PBS; Thermoscientific) was added to each dish. Culture dishes were kept at 4 °C for 30 min with gentle shaking. After PBS washing, cells were scraped and lysed in 100 μl M-PER buffer with proteinase inhibitors (Thermoscientific). One-hundred μg of total protein was pulled down with NeutrAvidin (Thermoscientific) beads according to the manufacturer’s protocol. Experiments were performed in biologic replicates.

### Luciferase assay

In a 96 well opaque plate (BD Falcon, Franklin Lakes, NJ), 2×10^4^ HEK293T/17 cells were transfected with 100 ng TCF reporter plasmid (Cignal Report, Qiagene) and 0.6 μl Surefect (Qiagene) with or without 100 ng empty vector, pcDNA3.1-LRP5.WT-MycHisC, pcDNA3.1-LRP5.Q89R-MycHisC, or 10 ng pcDNA3.1-DKK1 vectors. Twenty-four hours after transfection, cells were treated with or without 50 or 100 ng/ml mouse recombinant Wnt3a (R&D systems, Minneapolis, MN) in Opti-MEM (Invitrogen) containing 0.5% fetal bovine serum (Invitrogen), 1% penicillin+streptomycin, and 2 mM glutamine (Thermoscientific). Eighteen hours later, Dual-Glow substrate (Promega) was added to each well, and the signal was detected with an M200 plate reader (Tecan, NC). Firefly luciferase activity was normalized to renilla luciferase activity. Experiments were performed in triplicates (n=3).

### Statistical analysis

Data were analyzed using the student’s *t*-test, with p<0.05 considered statistically significant.

## Results

### LRP5.Q89R did not change protein expression

**Figure 2:**
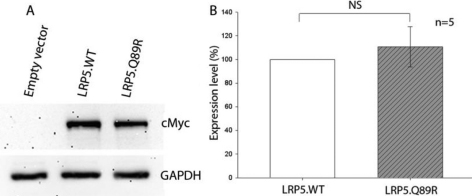
Expression of LRP5.WT and LRP5.Q89R was not different in transiently transfected HEK293T cells. HEK293T cells were transiently transfected with empty, LRP5.WT-MycHis or LRP5.Q89R-MycHis expression vector. Whole cell lysate was analyzed by western immunoblotting (**A**). LRP5.WT and LRP5.Q89R fusion proteins were detected with an anti-cMyc antibody. GADPH served as a loading control. (**B**) The expression levels of LRP5.WT and LRP5.Q89R were not statistically different. The optic density of LRP5 protein bands was first normalized to GAPDH and then normalized to LRP5.WT. Each column represents the mean±SD of normalized LRP5 expression of five samples (LRP5.WT 100%±0 versus LRP5.Q89R 111%±15%; n=5). NS: not significant, p>0.05.

LRP5 synthesis and/or degradation could be affected by the Q89R polymorphism. To answer this question, equal amounts of vectors encoding LRP5.WT-MycHisC or LRP5.Q89R-MycHisC fusion protein were transfected into HEK293T cells, and their expression levels were compared by western immunoblotting. The expression of LRP5 proteins were quantitated by densitometry. We did not find significant differences in the expression levels of LRP5.WT and LRP5.Q89R (Figure 2; *t*-test, n=5, p>0.05).

### LRP5.Q89R did not affect binding to MESD or protein trafficking

**Figure 3:**
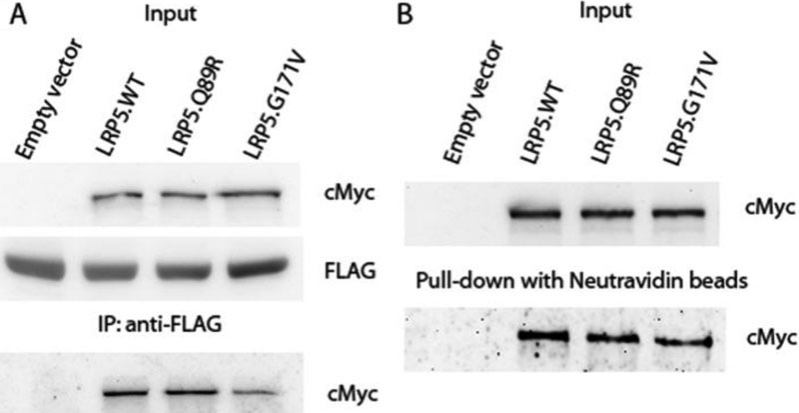
LRP5.Q89R did not affect binding to MESD or protein trafficking in HEK293T cells. LRP5.WT-MycHis, LRP5.Q89R-MycHis or LRP5.G171V was transiently expressed in HEK293T cells with (**A**) or without (**B**) MESD-FLAG by transfection. **A**: Co-IP of LRP5 and MESD protein complex. Input: LRP5.WT, LRP5.Q89R, LRP5G171V and MESD protein levels were studied by western immunoblotting with anti-cMyc or anti-FLAG antibodies, respectively. Whole cell lysate was immunoprecipitated with an anti-FLAG antibody (IP: anti-FLAG). The enriched protein was analyzed with SDS–PAGE and detected with anti-cMyc antibodies. **B**: Pull-down of cell surface LRP5 proteins. Proteins on the cell surface were labeled with biotin and pulled down with Neutravidin beads. Protein samples from input and after pull-down were subjected to western immunoblotting. LRP5.WT, LRP5.Q89R, and LRP5.G171V were probed with an anti-cMyc antibody. Experiments were performed in biologic replicates and representative data were shown.

MESD is a molecular chaperone involved in transporting LRP5 protein to the cell surface [25,27]. Mutations in the first β-propeller domain of LRP5 such as LRP5.G171V may alter its binding to MESD and lead to decreased amount of LRP5 on the cell surface [28]. We therefore studied whether LRP5.Q89R affects MESD binding. LRP5.WT-MycHis or LRP5.Q89R-MycHis were expressed together with MESD-FLAG in HEK293T cells as well as LRP5.G171V-MycHis as a technical control. LRP5 proteins were co-immunoprecipitated with an anti-FLAG antibody. LRP5.Q89R was not different from LRP5.WT in binding to MESD, but as expected, the binding of G171V was compromised (Figure 3A).

Although LRP5.Q89R does not alter MESD binding, it is still possible that there is less LRP5.Q89R expressed on the cell surface. Therefore, we overexpressed LRP5.WT-MycHis, LRP5.Q89R-MycHis or LRP5.G171V-MycHisC in HEK293T cells, labeled surface proteins with biotin and pulled them down with Neutravidin beads. We found equal amounts of LRP5.WT and LRP5.Q89R on the cell surface but less LRP5.G171V (Figure 3B).

### LRP5.Q89R did not affect the canonical Wnt signaling pathway

**Figure 4:**
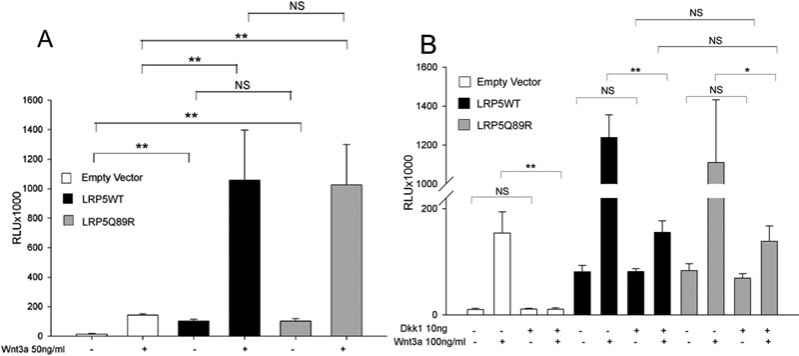
The canonical Wnt signaling activity in HEK293T cells after LRP5 overexpression. HEK293T cells were transiently transfected with TCF luciferase plasmids and empty, LRP5.WT, or LRP5.Q89R expression vectors. **A**: Wnt signaling activity after treatment with or without 50 ng/ml recombinant Wnt3a. **B**: DKK1 inhibition of Wnt signaling. HEK293T cells were also co-transfected with or without 10 ng DKK1 expression vector. Cells were treated with or without 100 ng/ml recombinant Wnt3a. Each column represents the mean±SD of relative luminescence unit (RLU; ×1000) of three samples (n=3). *: p<0.05 **: p<0.01. NS: not significant, p>0.05.

We have shown that Q89R polymorphism did not change protein expression, molecular chaperone interaction or protein trafficking. To determine whether LRP5.Q89R is able to alter the canonical Wnt signaling, we overexpressed LRP5.WT or LRP5.Q89R in HEK293T cells, and the resulting canonical Wnt signaling activity was studied using a TCF luciferase assay. Overexpression of LRP5.WT or LRP5.Q89R enhanced basal and Wnt3a induced Wnt signaling activity, compared to the empty vector control (*t*-test, n=3, p<0.01; Figure 4A). However, the enhancement of Wnt signaling activity by LRP5.WT and LRP5.Q89R overexpression was similar (*t*-test, n=3, p>0.05; Figure 4A).

### LRP5.Q89R did not change its efficacy to DKK1-mediated inhibition

It has been reported that mutations in the first β-propeller domain of LRP5 may alter its sensitivity to DKK1-mediated inhibition [22]. To test whether this is true for LRP5.Q89R, we overexpressed LRP5 proteins with or without DKK1 in HEK293T cells and the canonical Wnt signaling activity was measured using the TCF luciferase assay (Figure 4B). Overexpression of DKK1 had little effect on basal Wnt signaling activity with or without increased expression of LRP5.WT or LRP5.Q89R (*t*-test, n=3, p>0.05). Wnt3a induced Wnt signaling activity in control, LRP5.WT and LRP5.Q89R cells was inhibited by DKK1 (*t*-test, n=3, p<0.05). However, the remaining Wnt signaling activity in LRP5.Q89R cells was similar to that of LRP5.WT cells (*t*-test, n=3, p>0.05), which suggests that DKK1 is equally efficacious in interacting with both proteins.

### LRP5.Q89R did not affect protein expression, binding to MESD or protein trafficking in GTM3 cells

**Figure 5:**
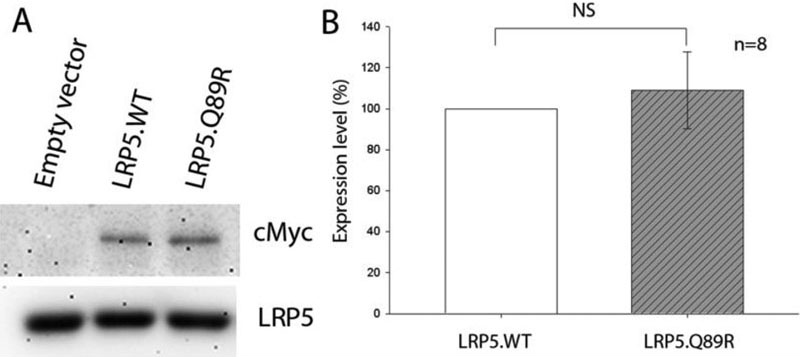
Expression of LRP5.WT and LRP5.Q89R was not different in transiently transfected GTM3 cells. GTM3 cells were transiently transfected with empty, LRP5.WT-MycHis or LRP5.Q89R-MycHis expression vector. Whole cell lysate was analyzed by western immunoblotting (**A**). LRP5.WT and LRP5.Q89R fusion proteins were detected with an anti-cMyc antibody. GADPH served as a loading control. **B**: The expression levels of LRP5.WT and LRP5.Q89R were not statistically different. The optic density of LRP5 protein bands was first normalized to GAPDH and then normalized to LRP5.WT. Each column represents the mean±SD of normalized LRP5 expression of eight samples (LRP5.WT 100%±0 versus LRP5.Q89R 109%±18%; n=8). NS: not significant, p>0.05.

**Figure 6:**
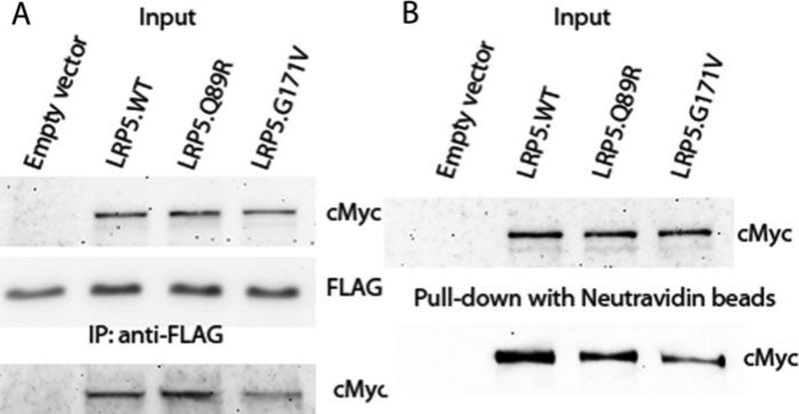
LRP5.Q89R did not affect binding to MESD or protein trafficking in GTM3 cells. LRP5.WT-MycHis, LRP5.Q89R-MycHis or LRP5.G171V was transiently expressed in GTM3 cells with (**A**) or without (**B**) MESD-FLAG by transfection. **A**: Co-IP of LRP5 and MESD protein complex. Input: LRP5.WT, LRP5.Q89R, LRP5G171V and MESD protein levels were studied by western immunoblotting with anti-cMyc or anti-FLAG antibodies, respectively. Whole cell lysate was immunoprecipitated with an anti-FLAG antibody (IP: anti-FLAG). The enriched protein was analyzed with SDS–PAGE and detected with anti-cMyc antibodies. **B**: Pull-down of cell surface LRP5 proteins. Proteins on the cell surface were labeled with biotin and pulled down with Neutravidin beads. Protein samples from input and after pull-down were subjected to Western immunoblotting. LRP5.WT, LRP5.Q89R and LRP5.G171V were probed with an anti-cMyc antibody. Experiments were performed in biologic replicates and representative data were shown.

The function of receptor proteins and cell signaling pathways depends on cell/tissue type. To test whether LRP5.Q89R behaves differently in the eye, we studied this polymorphism in a trabecular meshwork cell line GTM3. Similar to what we observed in HEK293T cells, LRP5.Q89R did not affect protein expression (Figure 5; *t*-test, n=8, p>0.05), binding to MESD (Figure 6A), or protein trafficking (Figure 6B) in GTM3 cells.

## Discussion

LRP5 is a key component of the canonical Wnt signaling pathway. Besides FEVR, LRP5 polymorphisms also are associated with bone diseases [8]. Many bone disease-associated LRP5 polymorphisms have been extensively studied and can cause two opposite phenotypes: high bone mass (HBM) and osteoporosis. LRP5 polymorphisms associated with HBM are mostly missense mutations found in the first β-propeller domain, while those associated with osteoporosis often cause frame shift or nonsense mutations [12].

How a single amino acid substitution in LRP5 affects Wnt signaling activity is still not fully understood. Zhang and colleagues [28] showed that the HBM-associated mutation LRP5.G171V disrupts LRP5 interaction with MESD, leading to decreased levels of surface LRP5. The authors proposed that this polymorphism enhanced Wnt signaling activity because decreased surface LRP5 reduces the target available for DKK1-mediated inhibition without compromising autocrine Wnt activity in osteocytes. However, Ai and colleagues [29] suggested that although LRP5 HBM mutations affect LRP5 expression on the cell surface, they are able to transduce Wnt signaling similar to LRP5.WT. They found HBM mutations bind less DKK1 and are therefore less inhibited. Other studies also suggested that the first β-propeller domain of LRP5 is critical for DKK1-mediated Wnt inhibition [22]. In addition to these two potential mechanisms, Semenov and He showed that LRP5.G171V has reduced binding to and inhibition by sclerostin (SOST), another Wnt inhibitor [26]. Interestingly, although DKK1 and SOST are both LRP5 inhibitors, they work independently and compete with each other [30].

In contrast to bone disease-associated LRP5 polymorphisms, eye disease-associated LRP5 polymorphisms have not been extensively studied. The LRP5 polymorphism LRP5.Q89R is particularly interesting to us because it has been associated with FEVR [19,20] as well as bone mass density [31], spinal osteoarthritis [32], and Type III hyperlipoproteinemia [33]. Structural analysis showed that LRP5.Q89R is located in the first β-propeller domain, where most of the HBM-associated polymorphisms reside. As discussed above, it seems that LRP5.Q89R may enhance Wnt signaling activity based on its location. Nevertheless, LRP5.Q89R-associated diseases all indicate that this polymorphism appears to impair the canonical Wnt signaling pathway. However, to our surprise in this study, we did not find any significant impact of the Q89R polymorphism on Wnt signaling activity in HEK293T or GTM3 cells. Another study of LRP5.Q89R in STF cells also reported that this polymorphism was able to respond to Norrin, a secreted Wnt pathway activator, provided that FZD4 was also expressed [20]. However, the effect of the Wnt signaling pathway is often cell/tissue-type dependent, so it is possible that LRP5.Q89R polymorphism may have altered functionality in other cell types. Furthermore, we cannot rule out the possibility that LRP5.Q89R may respond differently to other Wnt pathway activators/inhibitors, which may result in altered signal transduction. Finally, although LRP5.Q89R has been suggested to be “disease-associated” by genetic screening, it is still possible that this polymorphism has no functional consequence. Therefore, further studies of LRP5 polymorphisms are needed to elucidate their roles in various associated diseases.
